# Giving raw data a chance to talk: A demonstration of de-identified Pediatric Research Database (PRD) and exploratory analysis techniques for possible research cohort discovery and identifiable high risk factors for readmission

**DOI:** 10.1186/1471-2105-14-S17-A5

**Published:** 2013-10-22

**Authors:** Teeradache Viangteeravat, Eunice Y Huang, Grady Wade

**Affiliations:** 1 Biomedical Informatics Core, Children’s Foundation Research Institute, Memphis, TN, 38103, USA; 2 Department of Pediatrics, University of Tennessee Health Science Center, Memphis, TN, 38163, USA; 3 Decision Support, Le Bonheur Children’s Hospital, Memphis, TN, 38103, USA

## Background

Secondary use of large and open data sets provides researchers with an opportunity to address high impact questions that would otherwise be prohibitively expensive and time consuming. In spite of the data availability, often generating hypotheses from huge data sets is challenging, and lack of complex analysis of data might lead to weak hypotheses.

## Materials and methods

To overcome these issues and to assist researchers in building hypotheses from raw data, we are working on a methodology and informatics resource called the PRD, an acronym for “Pediatric Research Database.” The PRD is a de-identified database designed to make secondary use of rich data sources, i. e., electronic medical records (EMR). The development of visual analytics [1,2] makes the process of data elaboration, information gathering, knowledge generation, and complex information exploration transparent to tool users and provides researchers with the ability to sort and filter by various criteria, which can lead to strong, novel hypotheses. This database allows researchers to query large patient populations to identify small subsets based on certain inclusion and exclusion criteria. This not only permits users to detect expected events, such as might be predicted by models, but also helps users discover the unexpected – surprising anomalies, changes, patterns and relationships that are then examined and assessed to develop new insights. Only de-identified data are available from PRD to researchers. We maintain the identified data in a separate HIPAA class server room with very limited access. Individual-level data cannot be accessible without appropriate IRB approval. All potential re-identification attempts are protected by following the best practice de-identification process. All patients’ medical record number is replaced by an arbitrarily generated sequence number in order to prevent re-identification issues. To further protect patient re-identification, all patient count that is less than 10 would return “less than 10 patients” without any information. The outcome goal of the PRD is to facilitate clinical research and improve the health of children.

## References

[B1] Kamel Boulos M Viangteeravat T Anyanwu MN Ra Nagisetty V Kuscu E Web GIS in practice IX: a demonstration of geospatial visual analytics using Microsoft Live Labs Pivot technology and WHO mortality data Int J Health Geogr 2011 10 19 10.1186/1476-072X-10-19 21410968 PMC3068070

[B2] Flake G Is Pivot a turning point for Web exploration? http://www.ted.com/talks/gary_flake_is_pivot_a_turning_point_for_web_exploration.html (http://TED.com video - Feb/Mar 2010)

